# Electrochemical determination of calcium folinate in the presence of methotrexate and 5-fluorouracil using UiO-66/CdS composite modified screen-printed carbon electrode

**DOI:** 10.5599/admet.2897

**Published:** 2025-08-30

**Authors:** Fatma A. Khazaal, Qasim Mezban Salih, Raad Radi Karabat, Raed Muslim Mhaibes

**Affiliations:** 1 Department of Chemistry, College of Science, University of Wasit, Iraq; 2 Department of Pathological Analysis, College of Science, University of Wasit. Iraq; 3 Department of Medical Laboratory Techniques, Middle Technical University, Iraq; 4 Department of Biochemistry, College of Medicine, Misan University, Misan, Iraq

**Keywords:** Calcium folinate, methotrexate, 5-fluorouracil, electrochemical sensor, metal-organic frameworks, chemotherapy drugs

## Abstract

**Background and purpose:**

Calcium folinate is frequently co-administered with methotrexate and 5-fluorouracil in chemotherapeutic treatments to enhance therapeutic efficacy and reduce toxicity. Therefore, simple and accurate determination of calcium folinate in the presence of these agents is essential for therapeutic monitoring and pharmaceutical analysis.

**Experimental approach:**

The current work is focused on designing and developing a simple and sensitive modified screen-printed carbon electrode (SPCE)-based electrochemical sensor for the determination of calcium folinate in the presence of methotrexate and 5-fluorouracil. For this purpose, a nanostructure of UiO-66/CdS composite was prepared. Then, we used the prepared UiO-66/CdS composite to modify SPCE towards the development of an electrochemical sensing platform (UiO-66/ /CdS/SPCE).

**Key results:**

The cyclic voltammetry studies revealed that the UiO-66/CdS/SPCE could significantly improve the detection of calcium folinate with a higher response peak current at a lower overpotential compared to other SPCEs. Also, the UiO-66/CdS/SPCE sensor demonstrated a good ability in the quantitative determination of calcium folinate by the differential pulse voltammetry (DPV) method. Based on DPV measurements, a linearity plot was achieved for the concentration range of 0.1 to 300.0 μM calcium folinate with a limit of detection (LOD) of 0.04 μM and a high sensitivity of 0.0503 μA μM^-1^. Furthermore, the UiO-66/ /CdS/SPCE sensor showed three well-separated oxidation peaks for the simultaneous determination of calcium folinate, methotrexate, and 5-fluorouracil without interfering with each other. Finally, the UiO-66/CdS/SPCE sensor was used to detect calcium folinate, methotrexate, and 5-fluorouracil in real samples, and the values of recovery and relative standard deviation (RSD) were satisfactory.

**Conclusion:**

The results from this study show that the designed sensor can be used as an efficient and promising platform for the determination of these species.

## Introduction

Calcium folinate, alternatively referred to as Leucovorin calcium, is a vital medication with significant roles in diverse therapeutic contexts, particularly in cancer therapy, managing folate deficiency, and decreasing the unfavourable effects of some drugs [1]. Calcium folinate is a derivative of folic acid (vitamin B9) [2]. Folic acid plays a crucial role in the deoxyribonucleic acid (DNA) synthesis, red blood cell formation, and overall cell growth and division [3]. Unlike folic acid, calcium folinate does not need to be reduced by the enzyme dihydrofolate reductase (DHFR) to convert into tetrahydrofolate (active form of folate). One of the key clinical applications of calcium folinate is its role as a “rescue” agent in methotrexate therapy, a widely used chemotherapeutic medication. The anti-cancer effects of methotrexate occur due to its ability to inhibit the DHFR enzyme [4]. By inhibiting this process, methotrexate disrupts the formation of tetrahydrofolate, resulting in a depletion of folate within the cells. This interference with folate metabolism results in the toxic effects of methotrexate on rapidly dividing cells, including cancer cells and normal cells. By supplying a direct source of tetrahydrofolate, calcium folinate replenishes the levels of necessary folate for normal cells, thereby reducing the side effects of methotrexate [5,6]. Also, calcium folinate enhances the therapeutic effect of another chemotherapy medication, 5-fluorouracil [7,8]. A key mechanism contributing to the anti-tumour activity of 5-fluorouracil is the inhibition of the enzyme thymidylate synthase [9]. Thymidylate synthase is essential to produce thymidine, a nucleotide necessary for DNA synthesis. Inhibition of this enzyme leads to a decrease in thymidine production, which impairs DNA synthesis and ultimately results in cell death. Further inhibiting this enzyme with calcium folinate enhances the antitumor activity of 5-fluorouracil. Considering the importance of calcium folinate in therapeutic contexts, it is important to develop an analytical method for its determination in the presence of methotrexate and 5-fluorouracil in various samples. The most commonly used analytical methods for analysis of these species are chromatography [10-12], capillary electrophoresis [13], and spectrophotometry [14]. Nonetheless, some of these detection methods are complex, require sample preparation, and take a long time to analyse, making it difficult to obtain accurate measurements. Electrochemical techniques are suitable for the sensitive and efficient determination of electroactive substances due to their benefits, including cost-effectiveness in equipment, high sensitivity, rapid analysis, ease of use, and other advantageous features [1,6,15-17].

On the other hand, electrochemical sensors based on unmodified electrodes show limitations in the electroanalysis of target compounds, such as slow electron transfer rate, lower sensitivity, poor stability, limited detection range, surface fouling, and the requirement for a higher overpotential. Modified electrode-based electrochemical sensors are currently a key focus of research due to their enhanced response signals, improved sensitivity, and expanded detection range [18,19]. Based on the recent studies, the ability to engineer materials at the nanoscale opens up new possibilities for creating materials with interesting physico-chemical properties compared to their bulk counterparts. Due to the emergence of interesting properties in nanomaterials, their applications extend to various fields, including medicine, electronics, energy, catalysis, environmental remediation, materials science, and others [20-23]. Especially, the utilization of nanostructured materials for the modification of electrodes in voltammetric determinations of various compounds has been widely reported [24,25].

Metal-organic frameworks (MOFs) represent a novel category of crystalline porous materials comprising metal ions and organic ligands connected through coordination bonds. In comparison to conventional porous materials, MOFs exhibit distinctive features, including high adsorption ability, catalytic activity, a high surface area, tunable pore size, structural diversity, and customizable properties [26]. MOF-based materials (pristine MOFs, composites of MOFs, and derivatives of MOFs) have been extensively used in the areas of energy storage and conversion, sensing, catalysis, gas storage and separation, biomedicine, *etc.* [27-31]. Over the past decades, the utilization of MOFs in electrochemical sensing applications has become an interesting topic. The application of MOFs has been expanded to include the detection of various compounds in numerous areas, ranging from biomedical to environmental and food applications [32-38]. Moreover, the integration of UiO-66/CdS composite with screen-printed electrodes (SPEs) offers a promising platform for electrochemical sensing applications. SPEs are widely known as appropriate platforms for the advancement of sensing devices [39]. Their simple preparation and economical manufacturing enable the large-scale production of tailored electrode setups using various geometries, substrates, shapes, and sizes. Additionally, SPEs possess characteristics such as reduced sample preparation, disposability, rapid response, high reproducibility, and low sample requirements [40]. Furthermore, the use of SPEs allows for miniaturization of sensors for the determination of various compounds [41].

Considering this, a novel electrochemical sensing platform has been designed in this study for the determination of calcium folinate in the presence of methotrexate and 5-fluorouracil, based on a UiO-66/CdS composite-modified screen-printed carbon electrode (SPCE). The characterization of UiO-66/CdS composite and electrochemical performance of UiO-66/CdS/SPCE for the determination of calcium folinate were carried out. A sensitive voltammetric determination of calcium folinate was achieved using UiO-66/CdS/SPCE as the sensing platform. For calcium folinate determination, the designed sensor demonstrated good performance, with high sensitivity, a low limit of detection (LOD), and a wide linear range. Additionally, the simultaneous determination of calcium folinate, methotrexate, and 5-fluorouracil was successfully performed. Furthermore, the UiO-66/CdS/SPCE sensor was tested for its ability to analyse real samples, demonstrating its efficient potential.

## Experimental

### Chemicals

All the chemicals utilized in the experiments were of analytical reagent grade and were used as received without additional purifications. The solution used as a supporting electrolyte was phosphate buffer prepared with 0.1 M phosphoric acid, with the pH being adjusted to certain values through the addition of suitable amounts of sodium hydroxide solution. Deionized water was utilized throughout the experiments.

### Apparatus

A Metrohm Autolab was used in all the electrochemical studies and measurements. The commercially available SPCEs (Metrohm/DropSens (Spain)) were used. The counter electrode (CE) and working electrode (WE) consisted of carbon, and the pseudo-reference electrode was Ag.

### Synthesis of UiO-66/CdS composite

The synthesis of UiO-66 MOF was performed according to a method reported by Wu *et al.* [42] with some modifications. Initially, a mixture of 160 μmol of ZrCl4, 160 μmol of terephthalic acid (TPA), and 2.4 mL of acetic acid (AA) was added to N,N-dimethylformamide (DMF) (20 mL). The resulting mixture was sonicated for 40 minutes and then transferred into a Teflon-lined stainless-steel autoclave. The autoclave was placed in an oven and heated at 120 °C for 8 hours, then allowed to cool to room temperature. The obtained precipitates were collected by centrifugation, washed with distilled water and ethanol, and subsequently dried under vacuum. The synthesis of UiO-66/CdS composite was performed using the method described by Xu *et al.* [43] with slight modifications. For this purpose, 0.01 g of Cd(CH_3_COO)_2_·2H_2_O was dissolved in 10 mL of ethanol. Afterwards, 0.04 g of UiO-66 MOF was added to the solution, and the mixture was sonicated for 30 minutes. Subsequently, the suspension was heated at 80 °C for 10 minutes, after which 10 mL of an aqueous solution of thioacetamide (TAA, 0.0023 g) was added dropwise to the above solution under vigorous stirring, and the mixture was continued to be heated at 80 °C for an additional 30 minutes. Additionally, the prepared suspension was then heated further at 80 °C for 30 minutes. Finally, the prepared precipitates were collected by centrifugation, washed five times with distilled water and ethanol, and dried under vacuum.

### Preparation of modified SPCEs

For the preparation of UiO-66/CdS composite modified SPCE, 1.0 mg of the prepared UiO-66/CdS composite was weighed and dispersed into 1.0 mL deionized water, followed by ultrasonication process to obtain a homogeneous suspension (1.0 mg mL^-1^) of this nanostructure. Then, 4.0 μL of a well-dispersed suspension of UiO-66/CdS composite was dropped onto the surface of the SPCE and finally dried for 20 min under ambient conditions to evaporate the solvent from the suspension. This electrode was named UiO-66/CdS/SPCE.

## Results and discussion

### Characterization of UiO-66/CdS composite

**Figure 1.**
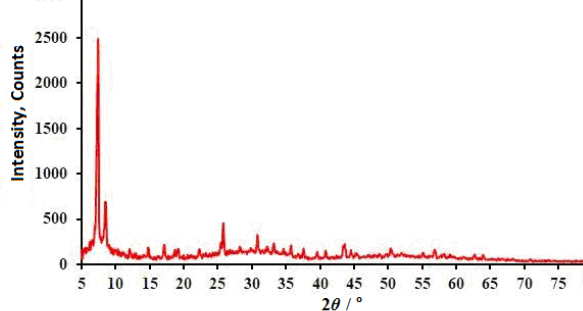
XRD pattern of UiO-66/CdS composite

The XRD pattern of UiO-66/CdS composite is exhibited in Figure 1. The peaks observed in the XRD pattern correspond to the crystalline structure of the UiO-66 MOF [42], while the absence of CdS peaks may be attributed to its low loading.

**Figure 2.**
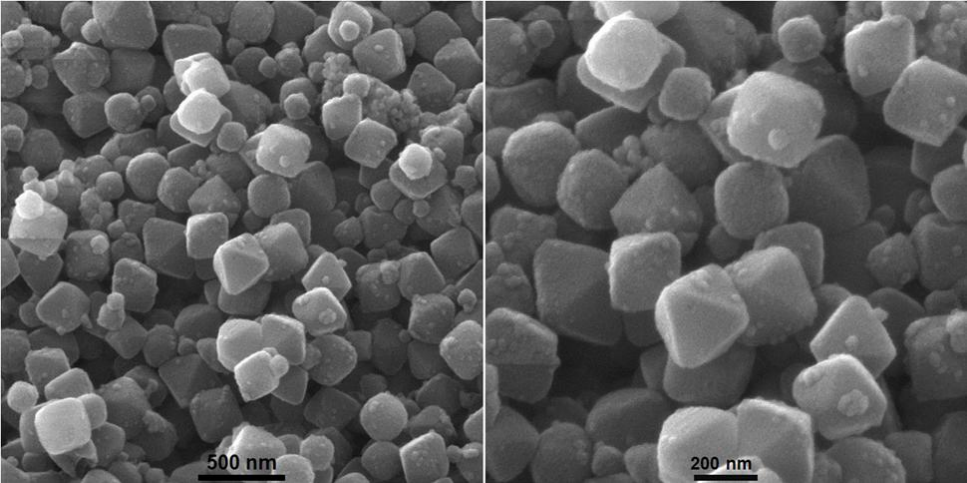
FE-SEM images of UiO-66/CdS composite at various magnifications

The FE-SEM technique was applied to investigate the structure and morphology of the prepared material. The morphological analysis of the as-prepared UiO-66/CdS composite by FE-SEM images is shown in Figure 2.

### Voltammetry response of UiO-66/CdS/SPCE towards calcium folinate compared to other SPCEs

Initial studies to investigate the influence of the pH of a 0.1 M phosphate buffer solution (PBS) on the DPV response of UiO-66/CdS/SPCE towards 50.0 μM calcium folinate were conducted at different pH values (pH 3.0 to 9.0). Based on the DPV experiments, the signal response of calcium folinate was significantly enhanced with increasing pH from 3.0 to 7.0. Nonetheless, a further increase in the pH caused a decrease in the signal response. The maximum signal response of calcium folinate was observed at pH 7.0 of PBS; therefore, it was used throughout the studies and measurements.

**Figure 3.**
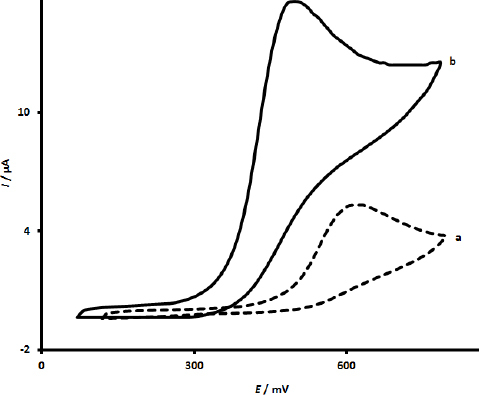
CV responses of unmodified SPCE (cyclic voltammogram a) and UiO-66/CdS/SPCE (cyclic voltammogram b) in pH 7.0 PBS containing 300.0 μM calcium folinate (The CVs recorded at a scan rate of 40 mV/s)

To evaluate the electrocatalytic performances, the cyclic voltammetry (CV) responses of unmodified SPCE and UiO-66/CdS/SPCE were carried out in pH 7.0 PBS containing 300.0 μM calcium folinate (Figure 3). The cyclic voltammogram of unmodified SPCE (a) presents a weak oxidation peak for calcium folinate with low current response (5.3 μA) at 625 mV. The cyclic voltammogram (b) demonstrates that the presence of UiO-66/CdS at the surface of SPCE decreases the potential to 500 mV and increases the current response (15.6 μA) of calcium folinate. This shows the important role of UiO-66/CdS in the oxidation process of calcium folinate. The observations of this investigation reveal that the presence of both UiO-66/CdS at the surface of SPCE considerably improves the response of SPCE, thereby enhancing the intensity of the oxidation peak of calcium folinate and shifting the oxidation peak position to more negative values.

### Effects of scan rate

**Figure 4.**
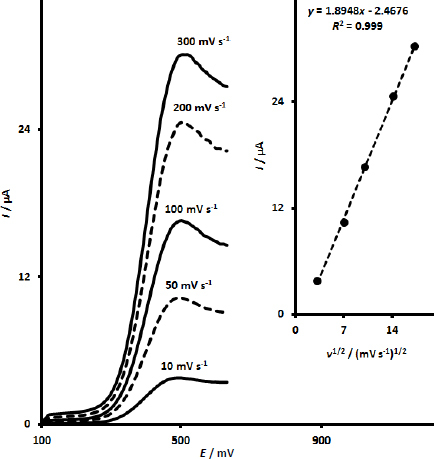
LSV responses of UiO-66-NH2 MOF/MWCNTs/SPCE in pH 7.0 PBS containing 200.0 μM calcium folinate at various scan rates. Inset: Linear relationship between the anodic peak currents of recorded cyclic voltammograms and *ν*^1/2^

To confirm whether the oxidation process of calcium folinate at the UiO-66/CdS/SPCE is adsorption or diffusion controlled, LSV studies were performed by varying scan rates from 10 to 300 mV s^-1^ in pH 7.0 PBS containing 200.0 μM calcium folinate. The corresponding cyclic voltammograms of this investigation are exhibited in Figure 4. With the increase in scan rate, the peak current of the corresponding cyclic voltammograms gradually increased. Furthermore, the cyclic voltammograms at UiO-66/CdS/SPCE exhibited a linear dependence between the anodic peak currents and the square root of the scan rate (*ν*^1/2^) (*I*_p_ = 1.8948*ν*^1/2^ - 2.4676 (*R*^2^ = 0.999)), as demonstrated in the inset of Figure 4. The observation of this linearity shows that the oxidation of calcium folinate at the UiO-66/CdS/SPCE is controlled by the diffusion process.

### Chronoamperometric investigations at UiO-66/CdS/SPCE

**Figure 5.**
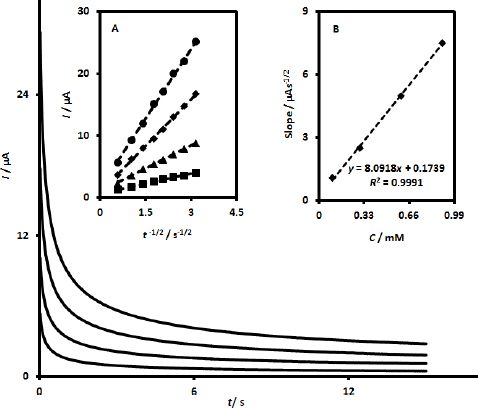
Chronoamperometric responses of UiO-66/CdS/SPCE in pH 7.0 PBS at concentrations 0.1, 0.3, 0.6 and 0.9 mM of calcium folinate (step potential at 550 mV *vs*. Ag/AgCl (3.0 M KCl)). Inset A: Cottrell plots of *I* against *t*^-1/2^ acquired from the chronoamperograms corresponding to a specific concentration of calcium folinate (the Cottrell plots were obtained within a certain period). Inset B: Linear dependence of slopes of Cottrell plots on the concentrations of calcium folinate

For the determination of the diffusion coefficient of calcium folinate in PBS at UiO-66/CdS/SPCE, chronoamperometric experiments were carried out at different concentrations of calcium folinate with an applied potential of 550 mV relative to the reference electrode. The corresponding chronoamperograms are exhibited in Figure 5.

**Figure 6.**
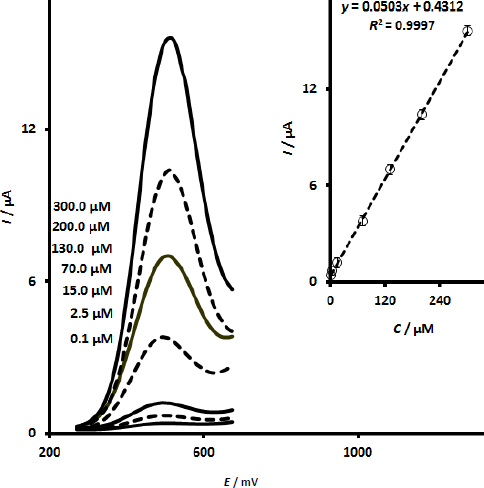
DPV responses of UiO-66/CdS/SPCE in pH 7.0 PBS for concentrations of calcium folinate. Inset: Linear relationship between the anodic peak currents of recorded differential pulse voltammograms and concentrations of calcium folinate

The data from chronoamperometry show that the increase in calcium folinate concentration is accompanied by an increase in current. By plotting current against *t*^-1/2^ for each of the recorded chronoamperograms associated with a specific concentration of calcium folinate, a Cottrell plot was obtained (Inset A, Figure 5). Then, a linear plot with a slope of 8.0918 μA s^1/2^ mM^-1^ was obtained by plotting the slopes of the Cottrell plots against concentrations of calcium folinate (Inset B, Figure 5). The diffusion coefficient of calcium folinate was found to be 5.6×10^-6^ cm^2^ s^-1^ from the slope of the resulting linear plot in Inset B of Figure 6 and employing the following Cottrell equation (*I* = *nFACD*^1/2^π^-1/2^*t*^-1/2^).

### Differential pulse voltammetry responses of UiO-66/CdS/SPCE in determining various concentrations of calcium folinate

Differential pulse voltammetry (DPV) is used for quantitative measurements of calcium folinate. The primary advantage of this method over methods such as CV is the low background current, which enhances the sensitivity of the measurements. For quantitative measurements, a series of DPV experiments was conducted in pH 7.0 PBS containing various concentrations of calcium folinate, and the differential pulse voltammograms are shown in Figure 6 (Step potential of 0.01 V and pulse amplitude of 0.025 V). As expected, the peak currents of voltammograms increased with the increase of calcium folinate concentration. The determination of calcium folinate at the UiO-66/CdS/SPCE showed a linear dependence between the peak current of the voltammograms and the calcium folinate concentration in the range of 0.1 to 300.0 μM. The calibration plot obtained for the UiO-66/CdS/SPCE sensor in determining calcium folinate is shown in Figure 6 (Inset). From the calibration plot, some important parameters will be obtained, including LOD, sensitivity, and linear response range. The calibration plot presented a wide linear response range of 0.1 to 300.0 μM with a high sensitivity of 0.0503 μA μM^-1^. The LOD of the methos was calculated as 0.04 μM (*S*/*N* = 3.0).

### DPV determination of calcium folinate in the presence of methotrexate and 5-fluorouracil

**Figure 7.**
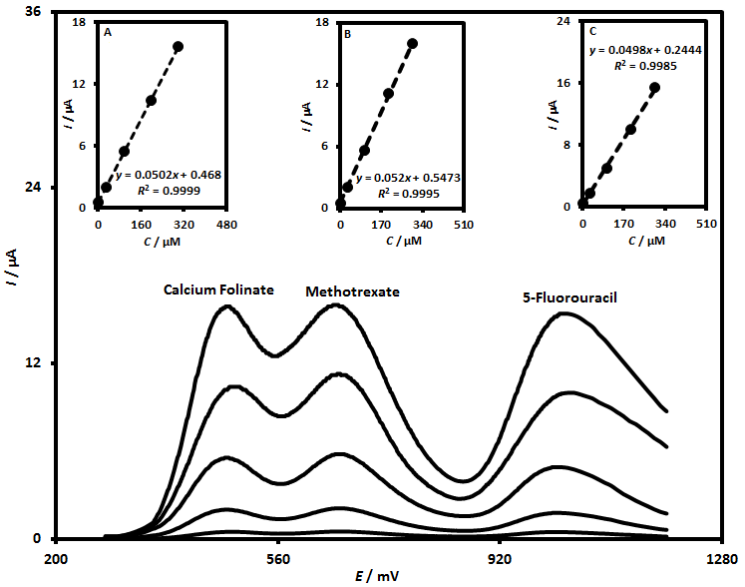
DPV responses of UiO-66/CdS/SPCE in pH 7.0 PBS at various concentrations of calcium folinate, methotrexate, and 5-fluorouracil (differential pulse voltammograms were recorded for 0.5+0.5+0.5 μM, 30.0+30.0+30.0 μM, 100.0+100.0+100.0 μM, 200.0+200.0+200.0 and 300.0+300.0+300.0 μM of calcium folinate, methotrexate and 5-fluorouracil, respectively). Corresponding calibration plots of calcium folinate (Inset A), methotrexate (Inset B) and 5-fluorouracil (Inset C)

The performance of the UiO-66/CdS/SPCE sensor for the determination of calcium folinate in the presence of methotrexate and 5-fluorouracil was investigated using the DPV method. DPV measurements were carried out under the optimized conditions by simultaneously changing the concentration of three species in pH 7.0 PBS. Figure 7 displays the differential pulse voltammograms obtained during this investigation (Step potential of 0.01 V and pulse amplitude of 0.025 V). As can be seen, three well-separated oxidation peaks for calcium folinate (*E*_pa_ = 485 mV), methotrexate (Epa = 665 mV), and 5-fluorouracil (*E*_pa_ = 1030 mV) were observed at the UiO-66/CdS/SPCE without interfering with each other. Additionally, it can be observed that the current responses of the target species (calcium folinate, methotrexate, and 5-fluorouracil) increased gradually with the increase in the corresponding target species concentration. Moreover, the current response of calcium folinate, methotrexate, and 5-fluorouracil demonstrated a suitable linear relationship with their concentrations in the ranges of 0.5 to 300.0 μM, respectively. The corresponding linear plots of calcium folinate, methotrexate, and 5-fluorouracil were exhibited in Figure 7A, 7B and 7C, respectively. The observation of these results revealed the feasibility of the designed electrochemical sensing platform for the simultaneous determination of calcium folinate, methotrexate, and 5-fluorouracil.

#### Application of UiO-66/CdS/SPCE in real sample analysis

The analysis of real samples is considered a key factor in investigating the performance of the designed sensors for practical applications. Therefore, the practical applicability of the UiO-66/CdS/SPCE sensor was evaluated by determining the amount of calcium folinate, methotrexate and 5-fluorouracil in their injection samples. Firstly, the levels of calcium folinate, methotrexate and 5-fluorouracil in these samples were determined. The recovery tests were subsequently conducted to validate the proposed method. The prepared samples were spiked with certain concentrations of calcium folinate, methotrexate, and 5-fluorouracil, and then the corresponding DPVs were recorded. The DPV response of UiO-66/CdS/SPCE sensor for calcium folinate was performed five times for each analysis, and the results are summarized in Table 1.

**Table 1.** The results for the determination of calcium folinate, methotrexate and 5-fluorouracil in real samples analysed by the suggested sensor (*n* = 5).

Sample	Concentrations, μM						Recovery, %			RS, %)		
	Spiked			Found								
	CF	MTX	5-FU	CF	MTX	5-FU	CF	MTX	5-FU	CF	MTX	5-FU
CF injection	0	0	0	3.9	-	-	-	-	-	3.4	-	-
	3.0	4.5	5.0	6.8	4.6	5.1	98.6	102.2	102.0	2.6	3.3	1.8
	5.0	6.5	8.0	9.1	6.3	7.9	102.2	96.9	98.7	1.9	2.5	3.4
MTX injection	0	0	0	-	2.4	-	-	-	-	-	2.5	-
	6.0	3.0	5.5	6.2	5.5	5.4	103.3	101.8	98.2	3.1	2.8	1.7
	8.0	5.0	7.5	7.7	7.2	7.6	96.2	97.3	101.3	1.9	3.4	2.7
5-FU injection	0	0	0	-	-	3.6	-	-	-	-	-	2.9
	6.5	5.0	3.0	6.4	5.1	6.7	98.5	102.0	101.5	2.3	1.8	3.3
	8.5	7.0	5.0	8.7	6.9	8.5	102.3	98.6	98.8	3.0	2.5	2.4

As can be seen, the recoveries were between 96.2 to 103.3 % with relative standard deviations (RSDs) lower than 3.4 %. The findings from this investigation demonstrate that the designed sensor may be a promising and valuable tool for determining calcium folinate, methotrexate and 5-fluorouracil in real samples.

## Conclusion

Herein, we designed an electrochemical sensor based on UiO-66/CdS/SPCE for the voltammetric determination of calcium folinate in the presence of methotrexate and 5-fluorouracil. From the CV studies, the modification of SPCE with the UiO-66/CdS composite effectively improved the electron transfer at the surface electrode, accelerated the response speed of the electrode, and enhanced the current response to calcium folinate. For the quantitative determination of calcium folinate, a good linear response was observed in the concentration range of 0.1 to 300.0 μM calcium folinate, with a high sensitivity of 0.0503 μA μM^-1^, as well as a low LOD of 0.04 μM. Also, the diffusion coefficient of calcium folinate was found to be 5.6M10^-6^ cm^2^ s^-1^. Based on the simultaneous determinations, when the UiO-66/CdS/SPCE sensor was applied to detect calcium folinate, methotrexate, and 5-fluorouracil, the oxidation peak of these compounds can be clearly separated. Furthermore, the sensing capabilities of calcium folinate, methotrexate, and 5-fluorouracil in the injection samples were effectively revealed by using the designed sensor. In brief, future directions will be guided by advanced techniques, including single-cell transcriptome analysis and AI and machine learning, to identify novel therapeutic targets and options for treating liver fibrosis.
